# Positional dyspnea due to excessive dynamic airway collapse: A case report

**DOI:** 10.1097/MD.0000000000036325

**Published:** 2023-12-15

**Authors:** Xiaoyan Sun, Zhenghui Cui, Yanxiong Mao

**Affiliations:** a Women’s Hospital School of Medicine Zhejiang University, Hangzhou, China; b Key Laboratory of Respiratory Disease of Zhejiang Province, Department of Respiratory and Critical Care Medicine, Second Affiliated Hospital of Zhejiang University School of Medicine, Hangzhou, China

**Keywords:** bronchoscopy, chronic obstructive pulmonary disease, dyspnea, excessive dynamic airway collapse

## Abstract

**Rationale:**

Excessive dynamic airway collapse (EDAC) is a form of dynamic central airway obstruction, with characteristic of excessive dynamic invagination of airway posterior wall membrane and structurally intact airway cartilage. We report a rare case of EDAC with a marked positional component.

**Patient concerns:**

A 73-year-old man was admitted to our hospital owing to dyspnea in right recumbent position (RRP). Also only in RRP, strong rhonchi was auscultated bilaterally through entire respiratory phase. He had gone through 3 episodes of resections on left lung due to hemoptysis caused by bronchiectasis, so he had only segment B1 + 2 and B3 left.

**Diagnoses:**

The spirometry results indicated that he had chronic obstructive pulmonary disease (COPD). The bronchoscopy revealed that in RRP, there was severe inward bulging of the posterior membrane of right main bronchus (RMB), which was worsened at expiratory phase. The EDAC of RMB was suspected, and was confirmed by an expiratory phase computed tomography (CT) in RRP. The EDAC was likely due to COPD, and the positional component was most likely to be caused by the removal of majority of his left lung.

**Interventions:**

Considering locality of EDAC and his overall stability, he was given a conservative approach. He was prescribed with budesonide/glycopyrrolate/formoterol for COPD and followed up.

**Outcomes:**

Two months later, the patient had relived dyspnea and weaker wheezing in RRP, and he had a good social and physical recovery.

**Lessons:**

Dyspnea may present as a diagnostic challenge, and it is rarely accompanied with a positional component. EDAC is an uncommon cause of dyspnea. This case illustrates the possible role of bronchoscopy and dynamic CT in dynamic evaluation of airway.

## 1. Introduction

Dyspnea is a common symptom, which present in many conditions, mainly cardiovascular and pulmonary diseases. Excessive dynamic airway collapse (EDAC) is a form of dynamic central airway obstruction, with characteristic of excessive dynamic invagination of airway posterior wall membrane and structurally intact airway cartilage.^[1]^ EDAC was traditionally referred to as the membranous type of tracheobronchomalacia. But now they are deemed to be 2 morphologically and physiologically distinct forms. The main symptoms of EDAC are nonspecific, including dyspnea, cough, sputum production, and hemoptysis.^[2,3]^ So EDAC could be the cause of dyspnea. But positional dyspnea due to EDAC has not been reported yet. Here, we report a rare case of EDAC with a marked positional component due to chronic obstructive pulmonary disease (COPD).

## 2. Case report

The patient was a 73-year-old man with comorbidity of hypertension. He was a nonsmoker. One year previously, he had been reporting dyspnea. The dyspnea was markedly worse when he lay on his right side. He could also hear loud wheezing from airway in right recumbent position (RRP). One week previously, he described exacerbated dyspnea and cough with purulent sputum, which was treated by cefuroxime for 6 days. He had improved cough and expectoration but not dyspnea, which led him to be admitted. He had reported recurrent hemoptysis due to bronchiectasis since teenage. He had gone through 3 episodes of resections on left lung due to hemoptysis, so he had only segment B1 + 2 and B3 left.

In physical examination, vital signs were normal. Surgery scars were detected. In RRP, strong rhonchi were auscultated bilaterally through entire respiratory phase. in left recumbent position or supine position, there was no rhonchi. The remainder of physical examination was unremarkable.

Spirometry revealed a forced expiratory volume in the first second (FEV1) of 39.7 % predictive, forced expiratory volume (FVC) of 45.7 % predictive and FEV1/FVC ratio of 66.16%, without bronchodilator reversibility. The spirometry results indicated that the patient had chronic obstructive pulmonary disease (COPD). A room air arterial blood gas revealed an PaO_2_ of 71 mm Hg and PaCO_2_ of 36 mm Hg.

**Figure 1.**
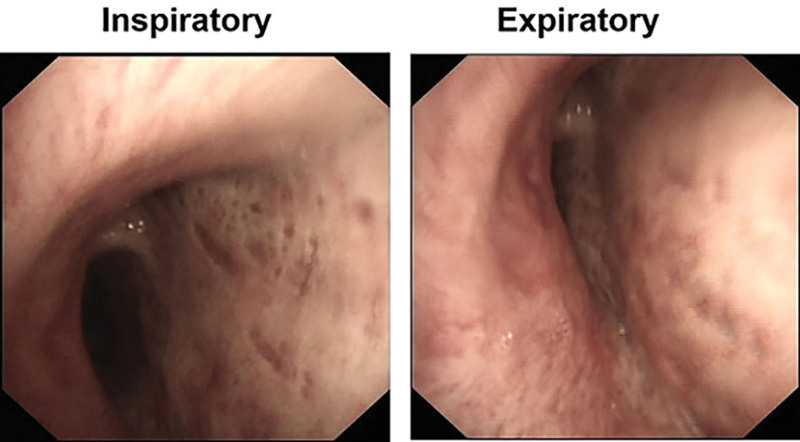
Bronchoscopy: The right main bronchus at the end of inspiratory and expiratory phase in right recumbent position.

Due to marked positional component of dyspnea, a chest computed tomography (CT) in RRP was ordered. To our surprise, no compression of the tracheobronchial tree was found. A flexible bronchoscopy was further carried out. During bronchoscopy, the patient was instructed to change his posture. In RRP, there was severe inward bulging of the posterior membrane of right main bronchus (RMB), which was worsened at expiratory phase (Fig. 1).

**Figure 2.**
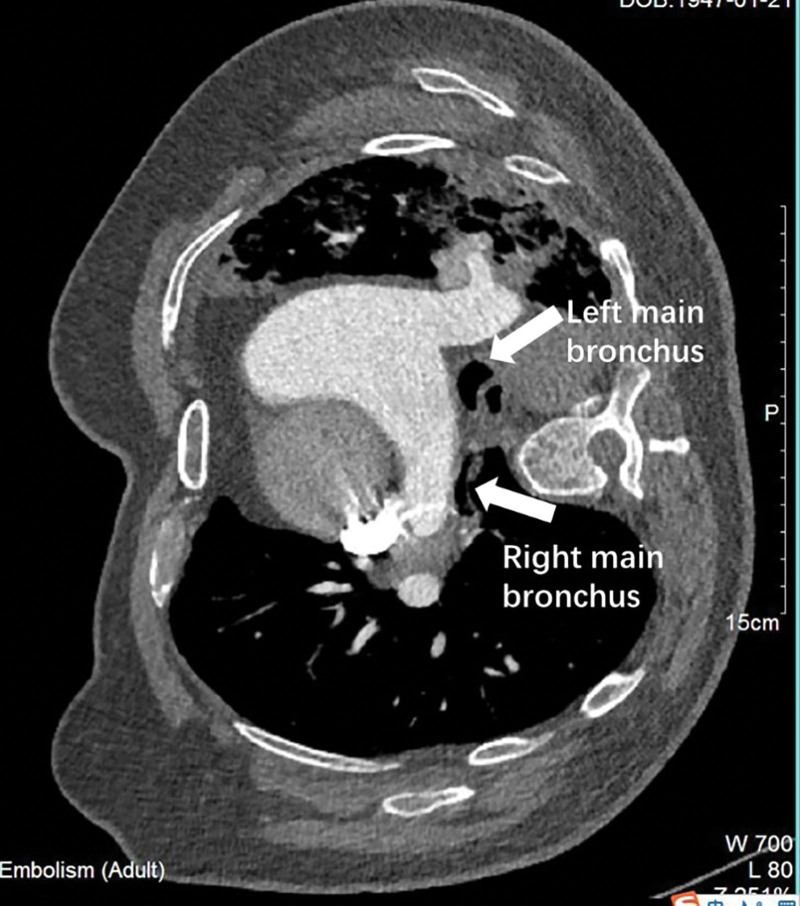
CT scan: The collapse of right main bronchus during an expiratory phase CT in right recumbent position. CT = computed tomography.

The EDAC of RMB was suspected, and was confirmed by an expiratory phase CT in RRP (Fig. 2). The reason why previous CT missed EDAC may be because CT is usually scanned at the end of inspiratory phase but collapse is significant at expiratory phase. The EDAC was likely due to COPD, and the positional component was most likely to be caused by the removal of majority of his left lung. Considering locality of EDAC and his overall stability, placement of endobronchial stents and surgical intervention was overruled in favor of a conservative approach to treat COPD. He was prescribed with budesonide/glycopyrrolate/formoterol (BREZTRI AEROSPHERE) for COPD and discharged.

Two months later, the patient had relived dyspnea and weaker wheezing in RRP, and he had a good social and physical recovery. Because of his improvement, we did not think it was necessary to order another bronchoscopy or dynamic CT. During the treatment, the patients reported no adverse events.

## 3. Discussion

To our knowledge, this was the first reported case of EDAC with a marked positional component. It was unclear whether his EDAC was congenital or acquired. He had a comorbidity of COPD. We suspected that his EDAC was likely due to COPD. In COPD, the decrease in transluminal pressure and weakening of the posterior smooth muscle membrane made the airways easily compressible and favor EDAC during cough or forced expiration. The peculiarity about our patient was the marked positional component of EDAC, and the cause was unclear. We suspected that the removal of majority of his left lung may contribute, but the exact mechanism warranted further study.

Previous studies showed that either EDAC or tracheobronchomalacia is present in 4% to 23% patients undergoing bronchoscopy for various indications.^[4]^ The causes of EDAC are varied, but can be broadly classified into congenital and acquired forms.^[5]^ The diagnosis of EDAC can be troublesome, given that a certain degree of collapse of the airway is physical during exhalation.^[6]^ Bronchoscopy is integral to establishing the diagnosis of EDAC, because it allows direct and dynamic visualization of the airways during breathing.^[4]^ CT can also be helpful in the diagnosis. Since the pathophysiology of this disease is dynamic, so too must be the CT. Dynamic CT allow physicians to compare the size of the airway lumen during inhalation and exhalation, thus detecting EDAC.^[1]^

Treatment depends on the severity of symptoms, degree and extent of collapse as well as etiology. Pharmacologic options are limited due to mechanical nature of EDAC. Nonpharmacologic options can range from positive pressure ventilation, to placement of endobronchial stents or surgical repair of the airways.^[7]^

The current study had 1 major limitation. The cause of his marked positional component of EDAC could not be clarified. We suspected that the removal of majority of his left lung due to recurrent hemoptysis caused by bronchiectasis may contribute. However, at present bronchial artery embolization represents the first-line treatment for hemoptysis caused by bronchiectasis, and the surgical removal of lungs has become a rare procedure. This indicate that it is very unlikely to have the similar patients. But there are large number of patients receiving surgical removal of lungs during to lung cancers. So maybe further study in such population could be helpful to explore the exact mechanism.

## 4. Conclusion

We reported the first case of positional dyspnea due to EDAC. Dyspnea may present as a diagnostic challenge, and it was rarely accompanied with a positional component. EDAC is an uncommon cause of dyspnea. This case illustrates the possible role of bronchoscopy or dynamic CT in dynamic evaluation of airway.

## Author contributions

**Data curation:** Xiaoyan Sun.

**Funding acquisition:** Xiaoyan Sun.

**Supervision:** Yanxiong Mao.

**Writing – original draft:** Xiaoyan Sun.

**Writing – review & editing:** Zhenghui Cui, Yaxniong Mao.
